# Schoolyard Biodiversity Determines Short-Term Recovery of Disturbed Skin Microbiota in Children

**DOI:** 10.1007/s00248-022-02052-2

**Published:** 2022-06-11

**Authors:** Jacob G. Mills, Caitlin A. Selway, Torsten Thomas, Laura S. Weyrich, Andrew J. Lowe

**Affiliations:** 1 School of Biological Sciences, The University of Adelaide, Kaurna Country, Adelaide, South Australia Australia; 2 Centre for Marine Science and Innovation, School of Biological, Environmental and Earth Sciences, University of New South Wales, Bidjigal Country, Sydney, Australia; 3 Department of Anthropology, The Pennsylvania State University, University Park, State College, PA USA

**Keywords:** Human microbiome, Urbanisation, Microbial ecology, Urban ecology, Lifestyle, Urban design

## Abstract

Creating biodiverse urban habitat has been proposed, with growing empirical support, as an intervention for increasing human microbial diversity and reducing associated diseases. However, ecological understanding of urban biodiversity interventions on human skin microbiota remains limited. Here, we experimentally test the hypotheses that disturbed skin microbiota recover better in outdoor schoolyard environments and that greater biodiversity provides a greater response. Repeating the experiment three times, we disturbed skin microbiota of fifty-seven healthy 10-to-11-year-old students with a skin swab (i.e., cleaning), then exposed them to one school environment—either a ‘classroom’ (*n* = 20), ‘sports field’ (*n* = 14), or biodiverse ‘forest’ (*n* = 23)—for 45 min. Another skin swab followed the exposure to compare ‘before’ and ‘after’ microbial communities. After 45 min, the disturbance immediately followed by outdoor exposure, especially the ‘forest’, had an enriching and diversifying effect on skin microbiota, while ‘classroom’ exposure homogenised inter-personal variability. Each effect compounded over consecutive days indicating longer-term exposure outcomes. The experimental disturbance also reduced the core skin microbiota, and only outdoor environments were able to replenish lost species richness to core membership (*n* species > 50% prevalent). Overall, we find that environmental setting, especially including biodiversity, is important in human microbiota recovery periods and that the outdoors provide resilience to skin communities. This work also has implications for the inclusion of short periods of outside or forest exposure in school scheduling. Future investigations of the health impacts of permanent urban biodiversity interventions are needed.

**Supplementary Information:**

The online version contains supplementary material available at 10.1007/s00248-022-02052-2.

## Introduction

There is increasing awareness that skin microbiotas are mechanistically important for human health, including immune and physiological responses [1–3]. Microbiota on the skin, and at other sites in the body, develop mostly through environmental factors and acquisition during early life following principles of community ecology and successional theory [2, 4–6]. These environmentally acquired microbiota, in particular co-evolved ‘old-friends’ microbiota [7], have the potential to shape life-long health trajectories [8].

However, there are everyday medical and lifestyle practices that can disturb the skin microbiota’s diversity and composition [e.g., hand sanitisation; 3, 9] and could contribute to the development and outcomes of skin diseases [2, 10]. Moreover, exposure to diverse environmental sources of microorganisms has become severely limited in modern cities, as humans spend more time indoors under clean or industrial conditions, which is linked to decreasing microbial diversity within the human body [11]. Overall, decreases in microbial exposure can have a number of potential health implications [12].

The recently proposed ‘microbiome rewilding hypothesis’ suggested that exposure to highly biodiverse urban environments may increase microbial diversity in humans [13, 14]. Recent experimental research showed that temporary biodiversity interventions, including importing forest soil rich in microbiota to day-care centres and playground sandpits, can increase microbial diversity on children’s skin and improve immune function [15, 16]. These results are akin to what is seen in children with more agrarian lifestyles [17, 18]. Therefore, simple urban biodiversity interventions plausibly constitute a positive health intervention. However, skin microbiotas have barely been examined in connection to permanent outdoor environments and outside play [19, 20], which is important for our understanding of the mechanistic interactions between a healthy skin microbiota and the broader environment.

Here, we aimed to understand the effect of permanent urban green spaces and relative biodiversity quality (e.g., functional diversity of ecological communities) on human skin microbial community enrichment after a disturbance common to modern behaviour—skin cleaning [3]. We tested two main hypotheses using ‘before’ and ‘after’ skin swabbing for microbiota of fifty-seven, 10–11-year-old, students around repeated, 45-min, exposures to indoor and outdoor schoolyard environments. Hypothesis one: after experimental skin community disturbance (the ‘before’ swab acted to clean the skin patch), exposure to green space will, within 45 min, restore diversity and change the composition and structure of skin microbial communities more than staying indoors; and hypothesis two: exposure to more biodiverse green space (forest) will have a greater effect on skin community diversity, composition, and structure than a less biodiverse green space (sports field).

## Methods

### Ethics

This project was done under ethics approval by The University of Adelaide’s Human Research Ethics Committee (approval number H-2019–064) and the South Australian Government’s Department for Education (approval number 2019–7,388,569).

### Metadata

Prior to participating in sampling, the school students and their parents or guardians were asked to complete a questionnaire about exclusion criteria: antibiotic use in the previous 6 months, allergies to sampling materials, and skin conditions.

### Design and Sampling

The study site was a primary school in Adelaide, Kaurna Country, South Australia, Australia. Participants were 10–11-year-old students at the school. We requested consent for 90 participants (80% recruitment, *n* = 72), and fifty-seven (*n* female = 25) passed our exclusion criteria.

Participants were already divided into three classes and had spent 9 months of the 2019 school year in this configuration before sampling in November. We assigned the classes to one of three treatment groups, or schoolyard environments—‘classroom’ (*n* = 20, *n* females = 10), ‘sports field’ (*n* = 14, *n* females = 7), or ‘forest’ (*n* = 23, *n* females = 8)—based on their teachers pre-existing proclivity to spend time in these environments to not disrupt their normal scheduling. We then followed a ‘before’ and ‘after’ exposure sampling regime, where the ‘before’ sampling also acted as a disturbance event to the skin microbiota and therefore provided pre-disturbance information. Participants were swabbed before and after a 45-min exposure to their assigned treatment environments.

The experiment was repeated over three consecutive days, 13^th^ November to 15^th^ November 2019, where we ran 1-h sessions from 9 to 10 am each day. After spending approximately 30–60 min in the classroom, the sessions started with a ‘before’ exposure sample in the classroom, followed by 45 min of standard school activities in their treatment environments followed by an ‘after’ exposure sample back in the classroom. The ‘before’ exposure samples on day one allowed us to test for differences in long-term microbiota divergence between groups over the course of the 2019 school year.

Samples were an epidermal skin swab collected by applying two drops of sterile saline solution (Reclens Saline Solution, Aaxis Pacific, Blacktown, Australia) to the inside of the participant’s left wrist as in Selway, et al. [19] followed by having them rub a nylon FLOQ swab (COPAN, Brescia, Italy) according to manufacturer’s instructions in an area 3 cm in diameter. The environments were also sampled for comparison to the human samples with swabs taken of the classroom tables (desks) and benches (sideboards) (*n* = 8), the sports field grass (*n* = 6), and the forest leaf surfaces (*n* = 4) and soil (*n* = 4). The swab tips of human and environmental samples were collected in 1 mL eNAT DNA stabilisation solution (COPAN, Brescia, Italy) and stored at − 20 °C until DNA extraction.

### DNA Extraction, PCR, Library Preparation, and Bioinformatics

DNA extractions were done across two different laboratories with different technicians due to COVID-19 restrictions. Samples were randomly assigned to the extraction labs. The first lab, Australian DNA Identification and Forensic Facility (ADIFF), randomly selected one sample from each sampling group (i.e., ‘treatment group’ × ‘exposure’ × ‘day’, or environmental sample) for each extraction batch done in that lab (*n* samples extracted at ADIFF = 140), and the remainder (*n* samples = 202) were sent to the second lab, the Australian Genome Research Facility (AGRF). DNA was extracted from human and environmental samples in both laboratories using the DNeasy PowerSoil Pro Kit (QIAGEN) as per the manufacturer’s instructions. Extraction blank controls (EBCs) were used for samples processed at ADIFF.

PCR amplification and sequencing of all samples were done by the AGRF. Bacterial 16S V3–V4 PCR amplicons were generated using the primers and conditions outlined in Table S1. Thermocycling was completed with an Applied Biosystem 384 Veriti and using Platinum SuperFi II master mix (Invitrogen, Australia) for the primary PCR. The first stage PCR was cleaned using magnetic beads (Beckman Coulter, SPRI), and samples were visualised on 2% Sybr Egel (Thermo-Fisher). A secondary PCR to index the amplicons was performed with the same polymerase master mix. The resulting amplicons were cleaned again using magnetic beads, quantified by fluorometry (Promega Quantifluor) and normalized. The equimolar pool of all amplicons was cleaned a final time using magnetic beads to concentrate the pool and then measured using a High-Sensitivity D1000 Tape on an Agilent 2200 TapeStation. The pool was diluted to 5 nM, and molarity was confirmed again using a Qubit High Sensitivity dsDNA assay (ThermoFisher). This was followed by sequencing on an Illumina MiSeq (San Diego, CA, USA) with a V3, 600 cycle kit (2 × 300 base pairs paired-end).

### Bioinformatics

Pre-processing was performed by AGRF using QIIME2 [21] version 2019.7. Samples were demultiplexed using Illumina scripts. Raw sequences were searched and trimmed for template-specific primers using Cutadapt with default quality settings [22]. Amplicon sequence variants (ASVs) were then generated at 240 bases using DADA2 [23]. Taxonomy was assigned to ASVs with the Silva 132 ‘sklearn’ classifier using a trained database for the 16S V3–V4 gene region [24].

We removed ASVs that were 100% biassed to one extraction lab or the other. Furthermore, we identified nine contaminant ASVs from non-template EBC and PCR controls using the prevalence method within the *decontam* package [v 1.8.0; 25] in R [v 4.0.0; 26] and with a threshold probability of 0.5. The nine identified contaminants were removed from all biological samples before downstream analysis. Additionally, 137 ASVs assigned to mitochondria, chloroplast, Archaea, or ‘unknown’ were removed, and 22 ASVs found in fewer than two biological samples in the dataset or with fewer than 9 reads [27] across all samples were also excluded. After pre-processing, there were 5412 ASVs with reference sequences and a total 18,835,659 sequences across 342 samples. Multiple sequence alignment for ASV sequences was constructed using the *msa* package [v 1.16.0; 28], and the *phangorn* package [v 2.5.5; 29] was used to build unrooted phylogenetic trees.

### Core-Community of Human Skin Samples

We determined core bacterial communities to test for experimental changes to the wrist community. To determine the core community, we divided the main dataset of human skin samples into six subsets based on the six exposure groups (i.e., treatment group by exposure combinations)—‘classroom before’, ‘classroom after’, ‘sports field before’, ‘sports field after’, ‘forest before’, and ‘forest after’—with the ‘subset_samples’ function of the *phyloseq* package [v 1.32.1; 30]. We then used the ‘ps_prune’ function of the *MicEco* package to keep only those ASVs that were present in at least 50% of the samples within each of these subsets. Once the ≥ 50% prevalent ASVs were identified, they were merged back into a single dataset using the ‘merge_phyloseq’ function of the *phyloseq* package. This process identified 39 ASVs as core to the skin samples of this project. We constructed an unrooted phylogenetic tree for the core community as above. We then merged the data of those 39 ASVs from the environmental samples into the human core community dataset for comparison between human and environmental sample types. From the 39 core ASVs, there were 6,451,217 total sequences across human and environmental samples.

### Statistics

All statistics were calculated in R [v 4.0.0; 26]. Three datasets were analysed, human skin communities (from skin swabs), core human skin communities (from skin swabs), and environmental communities (from soil, leaf surface, and classroom surface swabs).

Before alpha diversity was calculated, the filtered ASV datasets were rarefied to 3124 reads for the human skin communities, 1103 reads for the core human skin communities, and 10,933 reads for the environmental communities with the ‘rarefy_even_depth’ function of the *phyloseq* package [v 1.32.1; 30]. Alpha diversity was calculated as observed ASV richness and Shannon’s diversity with the ‘estimate_richness’ function in *phyloseq*, and Faith’s phylogenetic diversity was calculated with the ‘pd’ function of the *picante* package [v 1.8.1; 31]. We converted Shannon’s diversity to effective number of ASVs by taking its exponent [32]. We used generalised linear mixed models (GLMMs) to test for difference in alpha diversity by crossing the fixed factors of ‘treatment group’, ‘exposure’, and ‘day’ and adding the random factors of ‘student id’, ‘student id interacting with day’ to account for repeat sampling, and ‘student id interacting with exposure’ to account for repeat exposures. GLMMs were done with the ‘glmer’ function of the *lme4* package [v 1.1–25; 33]. Distributions for the GLMMs were negative-binomial for observed ASV richness (count data) and Gamma for Faith’s phylogenetic diversity and effective number of ASVs (Shannon’s), which were positive, non-integer, and non-parametric. Main effects of the GLMMs were tested by Type II Wald Chi^2^ tests with the ‘Anova’ function of the *car* package [v 3.0–10; 34]. Pairwise comparisons of ‘treatment group’, ‘exposure’, and ‘day’ combinations were done by *z*-tests with Tukey *P* value adjustment with the ‘emmeans’ function of the *emmeans* package [v 1.6.0; 35].

Ordinations of beta diversity on the three datasets were done with the ‘ordinate’ function in *phyloseq*. Ordinations were based on unrarefied data in principal coordinates analysis (PCoA) with weighted-UniFrac and unweighted-UniFrac distance matrices [36]. We used PERMANOVA, with 999 iterations, with the ‘adonis’ function of the *vegan* package [v 2.5–6; 37] to test the model of ‘treatment group’ by ‘exposure’ by ‘day’. The combinations of the treatment groups with the exposure created the variable of exposure groups (i.e., treatment group level + exposure level, e.g., ‘classroom before’, ‘classroom after’). Pairwise comparisons between exposure groups (e.g., ‘forest before’ vs. ‘forest after’) were tested by PERMANOVA with 999 iterations with the ‘pairwise.adonis2’ function of the *pairwise.adonis* package [v 0.0.1; 38].

Shared ASVs between environmental, ‘before’ exposure, and ‘after’ exposure human samples were tallied using the ‘ps_venn’ function of the *MicEco* package [v 0.9.15; 39]. ASVs were plotted by sample type into detected/undetected heatmaps using the ‘pheatmap’ function of the *pheatmap* package [v 1.0.12; 40]. The heatmap cells were clustered based on Pearson correlation between rows and columns.

### Data Access

Raw sequence data is stored on the Sequence Read Archive server with BioProject ID: PRJNA738964. Phyloseq compatible metadata, ASV table, taxonomy table, ASV reference sequences, R scripts used, and ethics approvals can be found on Figshare with the https://doi.org/10.25909/14787867.

## Results

### Pre-Existing Differences Between Groups

**Fig. 1:**
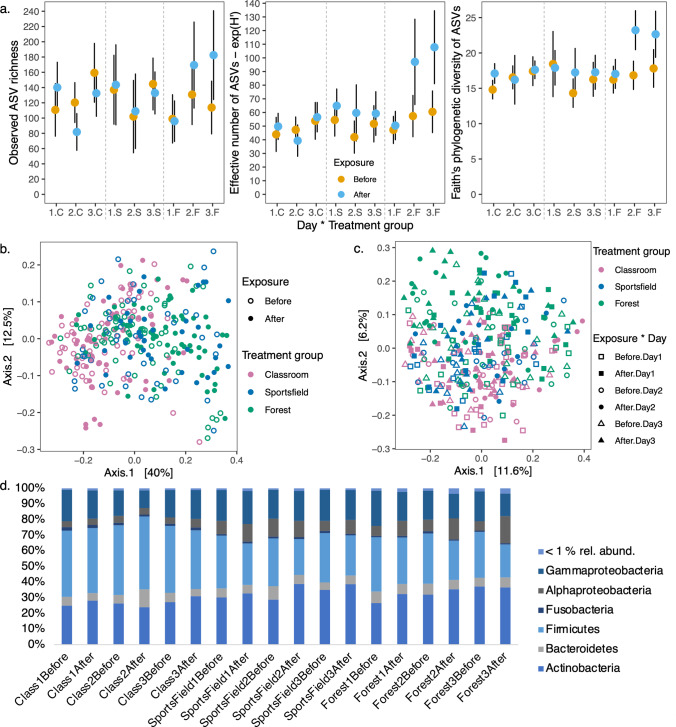
Bacterial ASV communities of children’s wrists ‘before’ and ‘after’ exposure to schoolyard environments repeatedly sampled across 3 days. **a.** Observed richness, effective number (exponent of Shannon’s diversity), and Faith’s phylogenetic diversity of ASVs are shown from the wrists of children exposed to three different schoolyard environments over three consecutive days. Points are means ± 95% confidence interval. Significantly different pairs are listed in Table 1. ‘1’, day 1; ‘2’, day 2; ‘3’, day 3; ‘C’, classroom; ‘S’, sports field; ‘F’, forest. **b. and c.** PCoA analyses of weighted-UniFrac and unweighted-UniFrac values, respectively, from all skin samples taken ‘before’ and ‘after’ outdoor exposure. Sampling ‘day’ is shown in the unweighted-UniFrac PCoA because it significantly interacted with ‘treatment group’ and ‘exposure’ in the PERMANOVA (Table S4). **d.** Relative abundance of bacterial phyla and proteobacterial classes in skin swabs. Sample names reflect the treatment group, sampling day, and exposure regime

Pre-existing differences between treatment groups (those exposed to classroom, sports field, or forest) were tested because each student group was together prior to this study for approximately 9 months of the 2019 school year [6]. There was no statistical support for differences in alpha diversity of bacterial communities between treatment groups prior to exposure on any sampling day (Fig. 1a; Table 1). However, we found statistically significant differences in bacterial community structure between ‘classroom’ and ‘sports field’, and ‘classroom’ and ‘forest’ groups prior to environmental exposure (weighted-UniFrac, Fig. 1b,c, Table 2). Further, all three treatment groups were significantly different in pre-exposure community compositions (unweighted UniFrac; Figure S1, Table 2). The ‘forest’ group had a significantly less diverse core community (i.e., ASVs present in at least 50% of samples within a group) prior to exposure on days ‘one’ and ‘three’ compared to equivalent ‘classroom’ group samples (Fig. 1a, Table S2). The structure of the core skin microbiota of the ‘classroom’ group was also significantly different to ‘sports field’ and ‘forest’ groups (Figure S2b and S2c, Table S3). Each treatment group was significantly different to each other in pre-exposure core community composition (Figure S2d and S2e, Table S3).

**Table 1:** Alpha diversity of bacterial (16S rRNA ASV) communities of student’s wrists before and after exposure to either forest, sports field, or classroom environments. EN is effective number of ASVs calculated as the exponent of Shannon’s diversity index. Faith’s PD is phylogenetic diversity of ASVs. Only showing significantly different pairs for at least one diversity index. Significance codes Pr(> Chi.^2^): ‘ns’ not significant; ‘º’ *P* < 0.10; ‘*’ *P* < 0.05; ‘**’ *P* < 0.01; ‘***’ *P* < 0.001

Descriptive statistics Treatment * exposure * day	Observed ASV richness			EN of ASVs (Shannon’s)			Faith’s PD of ASVs		
	Mean	95% CI	SE	Mean	95% CI	SE	Mean	95% CI	SE
Classroom before 1	110.7	34.4	16.4	43.9	12.5	6.0	14.8	1.3	0.6
Classroom after 1	140.1	33.0	15.8	49.8	9.5	4.6	17.1	1.4	0.7
Classroom before 2	120.4	26.1	12.5	47.3	9.4	4.5	16.5	1.6	0.8
Classroom after 2	81.8	24.0	11.5	39.3	11.4	5.5	16.2	3.4	1.6
Classroom before 3	159.3	38.5	18.4	53.8	13.5	6.4	17.4	2.0	1.0
Classroom after 3	132.6	30.3	14.5	56.5	10.8	5.1	17.6	1.2	0.6
Sports field before 1	137.1	45.1	20.9	54.5	11.8	5.5	18.4	4.6	2.1
Sports field after 1	143.6	52.4	23.8	64.9	12.3	5.6	17.9	2.4	1.1
Sports field before 2	101.9	47.4	21.7	41.9	11.8	5.4	14.3	2.0	0.9
Sports field after 2	108.9	48.8	21.9	59.7	20.5	9.2	17.2	3.4	1.5
Sports field before 3	144.6	33.9	15.7	51.7	13.2	6.1	16.3	2.4	1.1
Sports field after 3	132.9	27.5	12.7	59.2	16.0	7.4	17.3	2.4	1.1
Forest before 1	99.0	31.6	15.2	47.2	9.9	4.8	16.2	1.9	0.9
Forest after 1	95.9	26.5	12.8	50.4	10.4	5.0	17.0	2.0	1.0
Forest before 2	130.8	39.3	18.8	57.4	15.1	7.2	16.8	2.0	1.0
Forest after 2	169.5	56.3	26.9	97.1	31.2	14.9	23.2	2.8	1.3
Forest before 3	113.8	34.5	16.5	60.5	15.2	7.3	17.8	2.7	1.3
Forest after 3	182.5	58.2	27.8	107.8	26.7	12.8	22.6	3.3	1.6
GLMM—Type II Wald Chi^2^ test ~ Treatment * exposure * day	Chi^2^	Pr(> Chi^2^)	Sig	Chi^2^	Pr(> Chi^2^)	Sig	Chi^2^	Pr(> Chi^2^)	Sig
Treatment group	0.08	0.963	ns	1.51	0.470	ns	1.55	0.462	ns
Exposure	1.58	0.209	ns	10.25	0.001	**	10.69	0.001	**
Day	9.95	0.007	**	4.18	0.124	ns	1.88	0.391	ns
Treatment * exposure	8.62	0.013	*	3.97	0.137	ns	2.99	0.224	ns
Treatment * day	27.52	< 0.001	***	11.09	0.026	*	7.21	0.125	ns
Exposure * day	0.62	0.734	ns	1.12	0.573	ns	1.37	0.503	ns
Treatment * exposure * day	16.00	0.003	**	10.61	0.031	*	17.85	0.001	**
Pairwise GLMM ~ Treatment * exposure * day	*z*-ratio	*P*	Sig	*z*-ratio	*P*	Sig	*z*-ratio	*P*	Sig
Classroom before 1 – forest after 2	− 2.47	0.551	ns	2.99	0.203	ns	3.78	0.018	*
Forest before 1 – forest after 2	− 4.23	0.003	**	3.53	0.044	*	3.42	0.063	º
Forest before 1 – forest after 3	− 3.88	0.013	*	3.29	0.092	º	2.58	0.469	ns
Forest after 1 – forest after 2	− 4.27	0.003	**	3.33	0.082	º	3.35	0.076	º
Forest after 1 – forest after 3	− 3.93	0.011	*	3.08	0.162	ns	2.38	0.623	ns
Sports field before 2 – forest after 2	− 2.99	0.205	ns	2.86	0.278	ns	3.91	0.011	*
Forest before 2 – forest after 2	− 2.00	0.869	ns	2.94	0.230	ns	4.03	0.007	**
Classroom after 2 – forest after 2	− 3.99	0.008	**	3.55	0.041	*	3.14	0.141	ns
Classroom after 2 – classroom before 3	− 4.23	0.003	**	1.90	0.911	ns	0.88	1.000	ns
Classroom after 2 – forest after 3	− 3.68	0.026	*	3.35	0.078	º	2.34	0.651	ns
Forest before 3 – forest after 3	− 3.14	0.140	ns	3.56	0.040	*	2.93	0.237	ns

**Table 2:** Main (with homogeneity of dispersion tests, Disp.) and pairwise PERMANOVA on bacterial ASV community structure (Weighted-UniFrac) and composition (Unweighted-UniFrac) of student’s wrists before and after exposure to assigned school environments. Significance codes Pr(> F): ‘ns’ not significant; ‘º’ *P* < 0.10; ‘*’ *P* < 0.05; ‘**’ *P* < 0.01; ‘***’ *P* < 0.001

Main PERMANOVA Distance ~ Treatment*exposure*day		Weighted-UniFrac				Unweighted-UniFrac			
		*R*^2^	*F*	Pr(> *F*)	Disp.	*R*^2^	*F*	Pr(> *F*)	Disp.
Treatment	df_2,321_	0.09	16.29	***	**	0.02	3.94	***	***
Exposure	df_1,321_	0.02	7.09	***	ns	0.01	2.29	***	**
Day	df_2,321_	0.01	2.02	*	ns	0.01	1.57	**	**
Treatment*exposure	df_2,321_	0.01	1.94	*	º	0.01	1.65	**	º
Treatment*day	df_4,321_	0.01	1.11	ns		0.02	1.36	**	
Exposure*day	df_2,321_	0.01	1.00	ns		0.01	1.05	ns	
Treatment*exposure*day	df_4,321_	0.01	0.98	ns		0.01	1.22	*	**
Pairwise PERMANOVA Distance ~ Treatment*exposure		Weighted-UniFrac				Unweighted-UniFrac			
		*R*^2^	*F*	Pr(> *F*)		*R*^2^	*F*	Pr(> *F*)	
Forest before – classroom before	df_1,121_	0.06	8.14	***		0.02	2.44	***	
Forest before – sports field before	df_1,102_	0.01	0.96	ns		0.02	1.70	*	
Classroom before – sports field before	df_1,100_	0.06	6.82	***		0.01	1.48	*	
Forest after – forest before	df_1,123_	0.06	7.43	***		0.02	2.95	***	
Sports field after – sports field before	df_1,77_	0.03	2.37	*		0.02	1.52	*	
Classroom after – classroom before	df_1,119_	0.01	0.72	ns		0.01	0.98	ns	
Forest after – classroom after	df_1,121_	0.15	21.49	***		0.04	5.11	***	
Forest after – sports field after	df_1,98_	0.02	2.17	º		0.03	2.72	***	
Classroom after – sports field after	df_1,96_	0.13	13.65	***		0.02	2.38	***	
Forest after – classroom before	df_1,121_	0.19	28.20	***		0.05	6.03	***	
Forest after – sports field before	df_1,102_	0.05	5.49	***		0.03	3.64	***	
Classroom after – forest before	df_1,121_	0.05	5.77	***		0.02	1.99	***	
Classroom after – sports field before	df_1,100_	0.05	4.76	**		0.01	1.43	*	
Sports field after – forest before	df_1.98_	0.04	4.04	**		0.02	1.61	*	
Sports field after – classroom before	df_1,96_	0.16	18.18	***		0.03	2.58	***	

Therefore, for some microbial community measurements, there were unique signatures for each group’s skin microbiota prior to this experiment. These differences are despite all students being the same age, living within the same urban area, and attending the same school. Such uniqueness found between groups was consistent with another study suggesting that increasing time spent together, 9 months in this case, normalises within group skin microbiota [6]. Therefore, we accounted for pre-existing differences by focusing the remaining analysis on the impact of environmental exposure within each treatment group.

### Rapid Outdoor Enrichment of Disturbed Skin Microbiota

Environment plays a large deterministic role in the highly variable epidermal microbiota [41, 42]; therefore, we examined the microbial communities in each of the three exposure environments (i.e., swabbing classroom bench tops (sideboards) and tables (desks), sports field turf, forest soil, forest leaves) as potential determinants of student skin microbiota. We confirmed that forest soil and leaf bacterial alpha diversity were higher than classroom tables and sports field turf (Figure S3). Somewhat surprisingly, bacterial diversity of classroom bench tops was also quite high (Figure S3).

When we investigated the skin samples, we found that the ‘forest’ group had a significant increase in alpha diversity above pre-disturbance levels after 45-min exposures on days ‘two’ and ‘three’ (observed richness, effective number, and Faith’s phylogenetic diversity of ASVs; Fig. 1a, Table 1). The effect on the ‘forest’ group’s skin bacterial diversity also appeared to compound after each 45-min exposure from days one to three (Fig. 1a, Table 1). This was not so for the ‘classroom’ nor ‘sports field’ groups. Furthermore, only the ‘forest’ and ‘sports field’ groups had statistically significant changes in their bacterial community structure (weighted UniFrac) after exposure to their respective environments (Fig. 1b,c, Table 2). Composition (unweighted UniFrac) of skin bacterial communities significantly interacted between the factors ‘treatment group’, ‘exposure’, and ‘day’ (Figure S1, Table 2), as the composition turned over significantly for the ‘forest’ and ‘sports field’ groups after each exposure (Figure S1, Table 2). We also found that only the ‘classroom’ group’s skin microbiota became less variable between individuals over repeated days (dispersion test, *P* < 0.05, Table S4).

Several higher taxonomic groups changed in relative abundance between sampling groups in conjunction with the changing ASV compositions, reflecting changing dominance patterns due to the experimental design. Firmicutes noticeably reduced in relative abundance, by between 5.1 and 8.3% in after exposure samples (compared with ‘before’), each day for groups that went outside, while the classroom group ranged between + 1.8 and − 5.1% after exposure (Fig. 1d, Table S5). The alpha-Proteobacteria ranged in relative abundance in the forest groups after exposure samples from + 3.3 to + 11.3% compared to before exposure, while the sports field group ranged between − 0.7 and + 2.7%, and the classroom group was between − 0.1 and + 1.5%. Meanwhile, the gamma-Proteobacteria were marginally less dominant in after exposure samples across the board, ranging from + 1.5 to − 5.2% when compared to before exposure samples (Fig. 1d, Table S5). Furthermore, the Actinobacteria steadily increased across the sampling days for the classroom, sports field, and forest groups, ranging from 24.9% relative abundance on day one to 31.0% by day three, 30.1% on day one to 38.7% by day three, and 26.6% on day one to 37.3% by day three, respectively (Fig. 1d, Table S5).

**Fig. 2:**
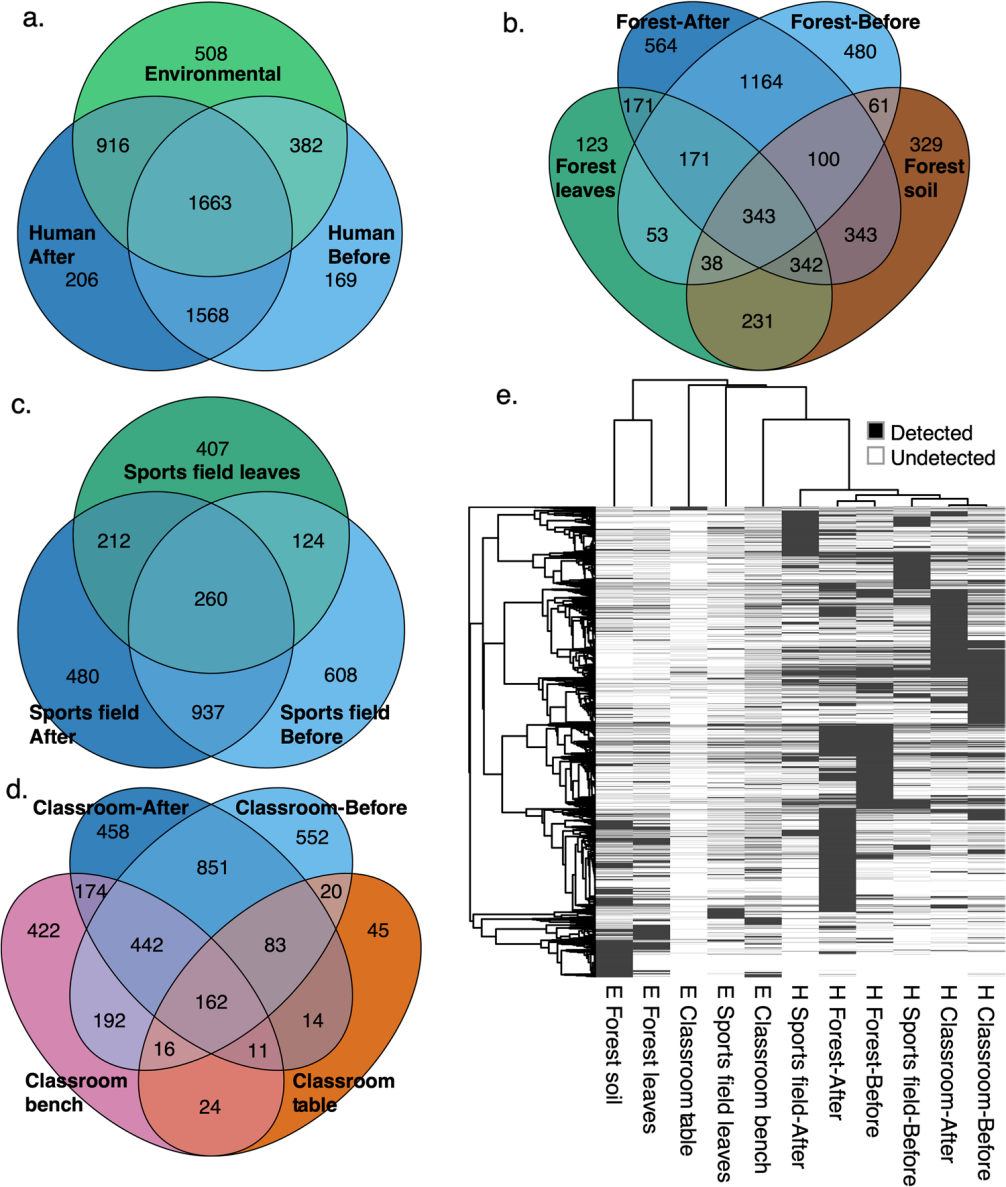
Shared and unshared bacterial community ASVs between human samples and environmental samples. **a.** Total shared and unshared bacterial ASVs between environmental samples and human samples collected ‘before’ and ‘after’ exposure. **b.** Total shared and unshared bacterial ASVs between the ‘forest’ environmental samples (soil and leaf surfaces) and human samples from the ‘forest’ treatment group collected ‘before’ and ‘after’ exposure. **c.** Total shared and unshared bacterial ASVs between the ‘sports field’ environmental samples (leaf surfaces) and human samples from the ‘sports field’ treatment group collected ‘before’ and ‘after’ exposure. **d.** Total shared and unshared bacterial ASVs between the ‘classroom’ environmental samples (bench tops and work tables) and human samples from the ‘classroom’ treatment group collected ‘before’ and ‘after’ exposure. **e.** Heatmap of detected community bacterial ASVs by sample type with clustering representing Pearson correlation between columns (samples) and between rows (ASVs). H and E on the x-axis represent human and environmental sample types, respectively

We next explored which ASVs were shared between human and environmental samples as representatives of microorganisms that were transferred onto the skin during these exposures. Overall, of the 1122 ASVs present in only ‘after’ exposure skin samples, 81.6% (916) were found in environmental samples (Fig. 2a). The ‘forest’ group lost 26.2% (632 of 2410) of their ‘before’ exposure ASVs but gained 1420, totalling 3198 ASVs ‘after’ exposure. For the ‘forest’ group, 171 of the acquired ASVs were found on forest leaf surfaces, 343 in forest soil, and 342 on both, while 564 had an unknown origin during environmental exposure (Fig. 2b). In contrast, the ‘sports field’ group lost 38.5% (742 of 1929) of their ‘before’ exposure ASVs and gained 692 during exposure, totalling 1889 ASVs after exposure, with 212 found on the sports field leaves (turf grass) and 480 of unknown origin (Fig. 2c). The ‘classroom’ group lost 780 of 2318 (33.6%) ASVs from their ‘before’ exposure samples and gained 657 ASVs (totalling 2195 ASVs ‘after’ exposure) found on bench tops (174), or tables (14), both (11), or unknown origin (458) (Fig. 2d). Overall, during exposure, the ‘forest’ group more than doubled ASVs that were lost through disturbance, while the ‘sports field’ and ‘classroom’ groups had net losses.

When looking at the total pool of samples (i.e., Fig. 2a), we found 206 skin associated ASVs from an unknown environmental origin in ‘after’ exposure samples. This was much lower than the ‘unknown origin’ fraction of skin microbiota in ‘after’ exposure samples when comparing within group skin samples to those of their treatment environment only (i.e., Fig. 2b–d). For example, the ‘forest’ group had 564 ASVs of unknown origin when compared only to forest soil and leaf samples (Fig. 2b) and not compared to classroom or sports field environmental samples, as compared to 206 when all samples were pooled. This finding means that many microbes were found in samples of students between exposure groups but not in all environmental samples between school exposure environments.

### Common Epidermal Microbiota Fluctuate with Disturbance

In total, there were thirty-nine core ASVs (0.96% of 4058 total human skin ASVs) that accounted for 35.6% (6,330,543 of 17,783,080) of total human skin ASV sequences. Observed ASV richness of the ‘classroom’ group’s core community was significantly reduced after disturbance and exposure on days ‘two’ and ‘three’ (Figure S2a), while effective number and Faith’s phylogenetic diversity of ASVs were not significantly different (Table S2). Therefore, minimal classroom recovery of common bacterial associate richness was possible. In contrast, core richness was not different for the ‘sports field’ nor ‘forest’ groups (Figure S2a) ‘after’ exposure, nor across days. However, change of core bacterial community structure was strongest in the ‘forest’ group when comparing ‘before’ and ‘after’ exposure samples (*R*^2^ = 0.33) relative to the ‘sports field’ (*R*^2^ = 0.26) and ‘classroom’ (*R*^2^ = 0.24) groups (Figure S2c, Table S3). Therefore, ASV turnover in the ‘forest’ and ‘sports field’ groups core microbiota was buffering decreases in diversity within 45 min of skin disturbance. We also note that core composition on the skin of the ‘classroom’ (*R*^2^ = 0.01, *P* > 0.05, Figure S2e, Table S3) and ‘sports field’ (*R*^2^ = 0.04, *P* > 0.05) groups did not significantly change between ‘before’ and ‘after’ exposure, while the ‘forest’ group did (*R*^2^ = 0.07, *P* < 0.05).

Lastly, we investigated the community turnover of ASVs into the core microbiota from schoolyard exposure. We first identified lost core community ASVs. Of the 29 original core ASVs across the three groups, eight were lost, while ten were gained during exposure to treatment environments (Figure S4a). In the ‘forest’ group, seven core ASVs were gained from the environment (6 from forest soil and leaves; 1 on leaves only), after losing seven from pre-disturbance (Figure S4b). The ‘sports field’ group gained seven new core ASVs (6 from sports field leaves; 1 unknown origin), after losing nine (Figure S4c). The ‘classroom’ group lost six core ASVs and gained two (Figure S4d) (1 on classroom benchtops, 1 on tables), with one of these of the potentially pathogenic group *Escherichia*-*Shigella *[43] (Figure S4e).

## Discussion

### Post-Disturbance Environment Determines Skin Microbial Heterogeneity

Increased diversity for the ‘forest’ groups skin microbiota over successive days shows that repeated and reasonably short exposures to more biodiverse areas could have longer-term diversifying effects, in-line with our hypotheses. However, our findings also unexpectedly showed that for 10–11-year-old children skin microbial diversity and variability between individuals within a group can reduce over time when skin communities are repeatedly disturbed by cleaning immediately followed by a short period of indoor time. In this regard, environment type immediately post-disturbance can be diversifying or homogenising for skin microbiota, and this may be the direct cause for skin microbiota homogenisation seen within groups over time [6]. We believe that ours is the first study to show such effects after just 45 min and thus extend previous studies showing that over hours [19] or weeks [16] change can occur to the skin microbiota according to exposure.

Age-related physiological changes in skin microbial habitats (due to puberty) and use of beauty products have previously been proposed to override environmental effects in homogenising adolescent (14-year-old) skin microbiota relative to younger children [44]. Here, we reproduced this homogenisation seen in adolescents in younger children by disturbing their skin microbiota and keeping them indoors immediately afterwards for a short period on each of 3 days. Younger children are often found playing outside, and it is usually older adolescents who tend to spend more time indoors for social reasons, because they have less energy for play and their school-based learning becomes more academic than experiential [i.e., more indoors; 45]. Therefore, we propose that environment is not overridden in adolescence, but that variability of environmental experience reduces between individuals (i.e., environment becomes more indoor). As such, environment is likely a key determining factor in age-related homogenisation of skin microbiota, as well as physiological and beautification factors. However, increasing environmental variability must be tested directly for increasing adolescent skin microbial diversity.

### Health-Associated Shifts in Dominant Skin Taxa

Shifts of dominant skin bacterial phyla and proteobacterial classes in this study align the indoor group with psoriatic compositions of dry skin sites while the outdoor groups tended towards healthier assemblages [46]. Here, the classroom group had consistently higher relative abundance of Firmicutes than Actinobacteria and Proteobacteria, whereas the outdoor groups had an opposing dominance structure. Such dominance structures have been found to associate with psoriatic lesions or healthy skin, respectively [47, 48]. Therefore, school-based, or more general social models of higher ratios of outdoor time for children, especially in biodiverse areas, may benefit sufferers of psoriasis.

Gamma-Proteobacteria have been found to be more dominant on the skin of children that are both exposed to more biodiversity and have higher measured immune function [16, 49]. However, we observed a general decrease of gamma-Proteobacteria in all groups from before to after exposure, indicating that recovery from skin microbiome disturbance may take longer than 45 min for these taxa. Such a finding indicates that continual disturbance to the skin may interrupt the ability of gamma-Proteobacteria to benefit the immune system, regardless of environment.

### Environmental Characteristics Influence Interactions

Previous studies have shown that floristically diverse urban green space soils are richer in microorganisms than less biodiverse spaces [50, 51]. Here, the school’s forest was generally richer in bacteria than the classroom and sports field and contributed to much stronger skin community recovery post-disturbance. However, while we found that high environmental microbial diversity can aid in increasing skin bacterial diversity after disturbance in a short amount of time, this was not always the case. Despite the high microbial diversity of the classroom bench tops the group exposed to the ‘classroom’ did not have an increase in skin diversity afterwards, highlighting that environmental microbial diversity probably interacts with environmental dynamics that vary by environment type. While we did not measure environmental dynamics directly, they may include lack of touch contact to some surfaces, such as students not touching high diversity classroom bench tops as much as students outside may directly touch soil or leaves [52]. Also, indoor and outdoor air-flow differences may contribute to aerial entrainment and redeposition differences of microorganisms onto skin. These factors may have reduced student interactions with high diversity bench tops around the classroom edges while increasing interaction in the forest where stronger recovery from disturbance was observed.

Vegetation contributes to the composition of downwind microbiotas, and those communities can be stratified by proximity to soil and wind speed [53–55]. Therefore, higher air-flow rate, vegetation complexity, and bare soil likely contributed to the ‘forest’ group’s increased skin diversity. Meanwhile, indoor microbiomes covary with outdoor air, ventilation source, air-flow rates, and skin of inhabitants [56, 57]. As such, indoor factors likely reduced environmental microbe-human interactions despite the presence of high diversity on some indoor surfaces. However, our evidence of cross-environmental sharing suggests that some ASVs from one environment are interacting with students in other environments (likely airborne as described above) or that we were not detecting some rarer bacteria in all samples [58]. Therefore, our results show strong human skin-environment interactions for microbial exchange and that adjacent environments may also be important microbial sources.

### Core Skin Microbiotas Under Disturbance

Core microbiota represent community members that are either temporally stable keystone species, functionally important facultative symbionts, or host-adapted obligate symbionts [59]. Here, we defined the core community as ASVs common to at least 50% of skin samples from ‘before’ or ‘after’ exposure within each treatment group. We found a small but highly abundant core that were vulnerable to disturbance and changed significantly according to disturbance and environmental exposure. However, while human skin core microbiota remains very poorly studied, common species are often functionally important [59], and further research into core skin microbiota is warranted, especially in the context of skin health.

## Conclusions

In line with our study hypotheses, exposure to green spaces enabled the recovery and enrichment of the skin microbial community within a short space of time (i.e., 45 min). Furthermore, exposure to a higher biodiversity setting (i.e., ‘forest’) provided a stronger effect than a lower biodiversity setting (i.e., ‘sports field’ or ‘classroom’). Unexpectedly, we also found that spending time indoors immediately post-disturbance can be homogenising of skin microbiota between individuals over time. This suggests that environment in the first 45 min post-disturbance is important for the state of the community in the longer term. Our findings provide further evidence that quality of biodiversity in our environments can enrich the human microbiota with diverse microbes and provide resilience to disturbance—the ability to maintain diversity in a dynamic ecology between environment, host, and microorganisms [60].

Diverse skin microbiotas have often been linked to positive health outcomes [2, 16–18, 49, 61, 62]; however, factors and mechanisms that underpin environmentally enabled resilience and how this may be related to long-term health trajectories require further investigation. Nevertheless, biodiversity interventions of urban green spaces have potential for positive effects on public health that can transcend socio-economic boundaries for health care [14]. If measures are not taken rapidly to prevent ‘green gentrification’—the increasing exclusivity of urban greening linked to socioeconomic status—then biodiversity interventions will not help those that are most in need of cost-effective primary health preventions [63]. The first place to start ensuring that people receive adequate access to diverse environmental microorganisms is in schools.

## Supplementary Information

Below is the link to the electronic supplementary material.

Supplementary file1 (DOCX 4536 KB)
