# Supplementary material for: Schoolyard Biodiversity Determines Short-Term Recovery of Disturbed Skin Microbiota in Children
Source: Microb Ecol. 2022 Jun 11;86(1):658–69. doi: 10.1007/s00248-022-02052-2 (PMC9188306; doi:10.1007/s00248-022-02052-2)
Supplement: Supplementary file 1 — Supplementary file1 (DOCX 4536 KB) [file 248_2022_2052_MOESM1_ESM.docx]

Supplementary Information: School yard biodiversity determines short-term recovery of disturbed skin microbiota in children

Jacob G. Mills^1^, Caitlin A. Selway^1^, Torsten Thomas^2^, Laura S. Weyrich^1,3^, Andrew J. Lowe^1^

^1^School of Biological Sciences, The University of Adelaide, South Australia, Australia

^2^Centre for Marine Science and Innovation, School of Biological, Environmental and Earth Sciences, University of New South Wales, Sydney, Australia

^3^Department of Anthropology, The Pennsylvania State University, University Park, USA

**Corresponding author**

Jacob G. Mills [millsj515@gmail.com](mailto:millsj515@gmail.com) or [jacob.mills@adelaide.edu.au](mailto:jacob.mills@adelaide.edu.au)

**Author contributions**

All authors contributed to conception and design. JM did the field work, part of the lab work, and statistics. All authors interpreted the results. JM wrote the first draft. All authors contributed to editing the manuscript.

**Table S1** PCR program and primers used at the Australian Genome Research Facility.

| **Target** | **Cycle** | **Initial** | | **Disassociate** | **Anneal** | **Extension** | **Finish** |
| --- | --- | --- | --- | --- | --- | --- | --- |
| 16S: V3 - V4 | 29 | 95ºC for 7 min | | 94ºC for 30 s | 50ºC for 60 s | 72ºC for 60 s | 72ºC for 7 min |
| **Primer sequence** | | |  | | | | |
| Forward Primer (341F) | | | CCTAYGGGRBGCASCAG | | | | |
| Reverse Primer (806R) | | | GGACTACNNGGGTATCTAAT | | | | |

**Table S2 Alpha diversity of core bacterial (16S rRNA ASV) communities of student’s wrists before and after exposure to either forest, sports field, or classroom environments.** EN is effective number of ASVs calculated as the exponent of Shannon’s diversity index. Faith’s PD is phylogenetic diversity of ASVs. Only showing significantly different pairs for at least one diversity index. Significance codes Pr(>Chi^2^): ‘ns’ not significant; ‘º’ P < 0.10; ‘*’ P < 0.05; ‘**’ P < 0.01; ‘***’ P < 0.001.

| Descriptive Statistics  Treatment * Exposure * Day | Observed ASV richness | | | | | | EN ASVs (Shannon’s) | | | | | | Faith’s PD of ASVs | | |
| --- | --- | --- | --- | --- | --- | --- | --- | --- | --- | --- | --- | --- | --- | --- | --- |
|  | Mean | | 95 % CI | | SE | | Mean | 95 % CI | | | SE | | Mean | 95 % CI | SE |
| Classroom Before 1 | 16.0 | | 1.6 | | 0.8 | | 7.8 | 1.1 | | | 0.5 | | 1.0 | 0.3 | 0.1 |
| Classroom After 1 | 15.0 | | 1.4 | | 0.7 | | 6.6 | 0.7 | | | 0.3 | | 0.8 | 0.3 | 0.1 |
| Classroom Before 2 | 17.1 | | 1.5 | | 0.7 | | 8.0 | 1.1 | | | 0.5 | | 1.0 | 0.3 | 0.1 |
| Classroom After 2 | 10.9 | | 2.3 | | 1.1 | | 5.8 | 1.4 | | | 0.7 | | 0.9 | 0.3 | 0.1 |
| Classroom Before 3 | 18.2 | | 1.9 | | 0.9 | | 8.2 | 1.2 | | | 0.6 | | 1.0 | 0.3 | 0.2 |
| Classroom After 3 | 15.0 | | 1.4 | | 0.7 | | 7.5 | 0.8 | | | 0.4 | | 0.9 | 0.3 | 0.1 |
| Sports field Before 1 | 16.0 | | 2.7 | | 1.2 | | 8.6 | 1.6 | | | 0.7 | | 1.3 | 0.3 | 0.1 |
| Sports field After 1 | 14.5 | | 1.7 | | 0.8 | | 8.5 | 1.6 | | | 0.7 | | 1.3 | 0.4 | 0.2 |
| Sports field Before 2 | 12.7 | | 3.2 | | 1.5 | | 6.9 | 1.4 | | | 0.6 | | 1.4 | 0.3 | 0.1 |
| Sports field After 2 | 13.8 | | 2.8 | | 1.2 | | 8.7 | 2.0 | | | 0.9 | | 1.3 | 0.2 | 0.1 |
| Sports field Before 3 | 16.4 | | 2.8 | | 1.3 | | 7.8 | 1.3 | | | 0.6 | | 1.1 | 0.3 | 0.1 |
| Sports field After 3 | 14.9 | | 1.5 | | 0.7 | | 8.0 | 0.7 | | | 0.3 | | 1.2 | 0.3 | 0.1 |
| Forest Before 1 | 11.5 | | 1.8 | | 0.9 | | 6.7 | 1.1 | | | 0.5 | | 1.3 | 0.2 | 0.1 |
| Forest After 1 | 9.7 | | 1.4 | | 0.7 | | 6.4 | 1.0 | | | 0.5 | | 1.3 | 0.2 | 0.1 |
| Forest Before 2 | 12.7 | | 2.3 | | 1.1 | | 7.1 | 1.2 | | | 0.6 | | 1.3 | 0.2 | 0.1 |
| Forest After 2 | 11.7 | | 2.1 | | 1.0 | | 7.0 | 1.3 | | | 0.6 | | 1.1 | 0.3 | 0.1 |
| Forest Before 3 | 11.6 | | 1.9 | | 0.9 | | 7.3 | 1.1 | | | 0.5 | | 1.1 | 0.3 | 0.1 |
| Forest After 3 | 12.5 | | 2.1 | | 1.0 | | 8.1 | 1.4 | | | 0.7 | | 1.1 | 0.2 | 0.1 |
| GLMM - Type II Wald Chi^2^ test  ~ Treatment * Exposure * Day | | Chi^2^ | | Pr(>Chi^2^) | | Sig. | Chi^2^ | | | Pr(>Chi^2^) | Sig. | Chi^2^ | | Pr(>Chi^2^) | Sig. |
| Treatment group | | 27.00 | | <0.001 | | *** | 1.07 | | | 0.587 | ns | 1.85 | | 0.396 | ns |
| Exposure | | 15.62 | | <0.001 | | *** | 0.77 | | | 0.381 | ns | 0.21 | | 0.648 | ns |
| Day | | 7.59 | | 0.022 | | * | 2.92 | | | 0.232 | ns | 0.16 | | 0.923 | ns |
| Treatment * Exposure | | 7.85 | | 0.020 | | * | 5.60 | | | 0.061 | º | 0.25 | | 0.883 | ns |
| Treatment * Day | | 10.56 | | 0.032 | | * | 3.04 | | | 0.551 | ns | 0.50 | | 0.974 | ns |
| Exposure * Day | | 1.70 | | 0.427 | | ns | 3.39 | | | 0.183 | ns | 0.08 | | 0.962 | ns |
| Treatment * Exposure * Day | | 14.42 | | 0.006 | | ** | 7.40 | | | 0.116 | ns | 0.19 | | 0.996 | ns |
| Pairwise GLMM  ~ Treatment * Exposure * Day | z-ratio | | P | | | Sig. | z-ratio | | P | | Sig. | | z-ratio | P | Sig. |
| Classroom Before 1 – Forest Before 1 | | 3.50 | 0.049 | | | * | -1.17 | | | 1.000 | ns | | 0.50 | 1.000 | ns |
| Classroom Before 1 – Forest After 1 | | 5.09 | < 0.001 | | | *** | -1.55 | | | 0.987 | ns | | 0.54 | 1.000 | ns |
| Classroom Before 1 – Classroom After 2 | | 4.21 | 0.003 | | | ** | -2.45 | | | 0.566 | ns | | -0.18 | 1.000 | ns |
| Sports field Before 1 – Forest After 1 | | 4.63 | < 0.001 | | | *** | -1.73 | | | 0.962 | ns | | 0.05 | 1.000 | ns |
| Forest Before 1 – Classroom Before 2 | | -4.20 | 0.004 | | | ** | 1.13 | | | 1.000 | ns | | -0.42 | 1.000 | ns |
| Forest Before 1 – Classroom Before 3 | | -4.94 | < 0.001 | | | *** | 1.52 | | | 0.990 | ns | | -0.42 | 1.000 | ns |
| Classroom After 1 – Forest After 1 | | 4.39 | 0.002 | | | ** | -0.45 | | | 1.000 | ns | | 1.15 | 1.000 | ns |
| Sports field After 1 – Forest After 1 | | 3.52 | 0.045 | | | * | -1.72 | | | 0.964 | ns | | 0.04 | 1.000 | ns |
| Forest After 1 – Classroom Before 2 | | -5.77 | < 0.001 | | | *** | 1.51 | | | 0.990 | ns | | -0.46 | 1.000 | ns |
| Forest After 1 – Classroom Before 3 | | -6.50 | < 0.001 | | | *** | 1.89 | | | 0.917 | ns | | -0.47 | 1.000 | ns |
| Forest After 1 – Sports field Before 3 | | -4.85 | < 0.001 | | | *** | 1.40 | | | 0.996 | ns | | -0.36 | 1.000 | ns |
| Forest After 1 – Classroom After 3 | | -4.36 | 0.002 | | | ** | 1.51 | | | 0.990 | ns | | -0.71 | 1.000 | ns |
| Forest After 1 – Sports field After 3 | | -3.94 | 0.010 | | | * | 1.67 | | | 0.972 | ns | | -0.22 | 1.000 | ns |
| Classroom Before 2 – Classroom After 2 | | 4.96 | < 0.001 | | | *** | -3.16 | | | 0.133 | ns | | -0.26 | 1.000 | ns |
| Classroom Before 2 – Forest After 2 | | 3.92 | 0.011 | | | * | -0.66 | | | 1.000 | ns | | 0.06 | 1.000 | ns |
| Classroom Before 2 – Forest Before 3 | | 3.97 | 0.009 | | | ** | -0.51 | | | 1.000 | ns | | 0.14 | 1.000 | ns |
| Forest Before 2 – Classroom Before 3 | | -3.85 | 0.014 | | | * | 0.83 | | | 1.000 | ns | | -0.42 | 1.000 | ns |
| Classroom After 2 – Classroom Before 3 | | -5.76 | < 0.001 | | | *** | 2.85 | | | 0.279 | ns | | 0.25 | 1.000 | ns |
| Classroom After 2 – Sports field Before 3 | | -3.60 | 0.035 | | | * | 1.88 | | | 0.920 | ns | | 0.29 | 1.000 | ns |
| Forest After 2 – Classroom Before 3 | | -4.64 | < 0.001 | | | *** | 1.04 | | | 1.000 | ns | | -0.07 | 1.000 | ns |
| Classroom Before 3 – Forest Before 3 | | 4.69 | < 0.001 | | | *** | -0.90 | | | 1.000 | ns | | 0.14 | 1.000 | ns |
| Classroom Before 3 – Forest After 3 | | 4.02 | 0.007 | | | ** | -0.32 | | | 1.000 | ns | | 0.13 | 1.000 | ns |

**Table S3** Main (with homogeneity of dispersion tests, Disp.) and pairwise PERMANOVA on core bacterial ASV community structure (Weighted-UniFrac) and composition (Unweighted-UniFrac) of student’s wrists before and after exposure to assigned school environments. Significance codes Pr(>F): ‘ns’ not significant; ‘º’ P < 0.10; ‘*’ P < 0.05; ‘**’ P < 0.01; ‘***’ P < 0.001.

| Main PERMANOVA  distance ~ Treatment*Exposure*Day |  | Weighted-UniFrac | | | | Unweighted-UniFrac | | | |
| --- | --- | --- | --- | --- | --- | --- | --- | --- | --- |
|  |  | R^2^ | F | Pr(>F) | Disp. | R^2^ | F | Pr(>F) | Disp. |
| Treatment | df_2,321_ | 0.11 | 19.59 | *** | º | 0.15 | 38.59 | *** | ** |
| Exposure | df_1,321_ | 0.02 | 7.04 | ** | ns | 0.10 | 51.25 | *** | * |
| Day | df_2,321_ | 0.01 | 1.18 | ns | ns | 0.01 | 1.58 | º | º |
| Treatment*Exposure | df_2,321_ | 0.02 | 3.03 | * | º | 0.15 | 38.46 | *** | ns |
| Treatment*Day | df_4,321_ | 0.01 | 0.63 | ns |  | 0.01 | 1.34 | ns |  |
| Exposure*Day | df_2,321_ | <0.01 | 0.86 | ns |  | 0.01 | 1.42 | ns |  |
| Treatment*Exposure*Day | df_4,321_ | <0.01 | 0.40 | ns |  | 0.01 | 1.06 | ns |  |
| Pairwise PERMANOVA  Distance ~ Treatment*Exposure |  | Weighted-UniFrac | | | | Unweighted-UniFrac | | | |
|  |  | R^2^ | F | Pr(>F) |  | R^2^ | F | Pr(>F) |  |
| Forest Before - Classroom Before | df_1,121_ | 0.06 | 8.22 | *** |  | 0.17 | 24.51 | *** |  |
| Forest Before - Sports field Before | df_1,102_ | 0.01 | 1.35 | ns |  | 0.16 | 18.85 | *** |  |
| Classroom Before - Sports field Before | df_1,100_ | 0.09 | 9.50 | *** |  | 0.24 | 30.62 | *** |  |
| Forest After – Forest Before | df_1,123_ | 0.07 | 9.84 | *** |  | 0.33 | 60.21 | *** |  |
| Sports field After - Sports field Before | df_1,77_ | 0.04 | 2.78 | º |  | 0.26 | 26.58 | *** |  |
| Classroom After - Classroom Before | df_1,119_ | 0.01 | 1.27 | ns |  | 0.24 | 37.12 | *** |  |
| Forest After – Classroom After | df_1,121_ | 0.18 | 26.10 | *** |  | 0.41 | 84.34 | *** |  |
| Forest After – Sports field After | df_1,98_ | 0.02 | 1.97 | ns |  | 0.25 | 31.91 | *** |  |
| Classroom After - Sports field After | df_1,96_ | 0.15 | 16.37 | *** |  | 0.29 | 39.17 | *** |  |
| Forest After – Classroom Before | df_1,121_ | 0.20 | 30.53 | *** |  | 0.46 | 101.4 | *** |  |
| Forest After – Sports field Before | df_1,102_ | 0.05 | 5.81 | ** |  | 0.32 | 47.78 | *** |  |
| Classroom After - Forest Before | df_1,121_ | 0.06 | 7.32 | ** |  | 0.08 | 11.19 | *** |  |
| Classroom After - Sports field Before | df_1,100_ | 0.08 | 8.24 | ** |  | 0.14 | 15.85 | *** |  |
| Sports field After - Forest Before | df_1.98_ | 0.05 | 4.96 | ** |  | 0.23 | 28.71 | *** |  |
| Sports field After - Classroom Before | df_1,96_ | 0.17 | 19.62 | *** |  | 0.31 | 43.51 | *** |  |

**Table S4** Permutation test (*n* permutations = 999) for homogeneity of multivariate dispersions on skin community structural (weighted UniFrac) and compositional (unweighted UniFrac) variability within treatment groups across the three days of experimental repetition. Significance codes Pr(>F): ‘ns’ not significant; ‘º’ P < 0.10; ‘*’ P < 0.05; ‘**’ P < 0.01; ‘***’ P < 0.001.

| Homogeneity of multivariate dispersions tests  ~ Day |  | Weighted-UniFrac | | | Unweighted-UniFrac | | |
| --- | --- | --- | --- | --- | --- | --- | --- |
|  | DF | F | Pr(>F) | Sig. | F | Pr(>F) | Sig. |
| Classroom | df_2,117_ | 2.29 | 0.098 | º | 4.60 | 0.015 | * |
| Sports field | df_2,75_ | 0.75 | 0.483 | ns | 0.82 | 0.440 | ns |
| Forest | df_2,121_ | 0.49 | 0.606 | ns | 1.06 | 0.345 | ns |

**Table S5** Relative abundance of dominant bacterial phyla and proteobacterial classes that were above 1 % relative abundance in skin swab samples, those below the threshold were grouped.

| Sample Group | Actinobacteria (%) | Bacteroidetes  (%) | Firmicutes  (%) | Fusobacteria  (%) | alpha-Proteobacteria (%) | gamma-Proteobacteria (%) | < 1 % rel. abund. (%) |
| --- | --- | --- | --- | --- | --- | --- | --- |
| Classroom Day1 Before | 24.9 | 5.6 | 42.4 | 2.3 | 3.7 | 20.2 | 1.0 |
| Classroom Day1 After | 28.1 | 4.8 | 41.5 | 2.1 | 4.1 | 18.1 | 1.2 |
| Classroom Day2 Before | 26.3 | 5.5 | 44.7 | 1.6 | 4.3 | 16.4 | 1.3 |
| Classroom Day2 After | 24.0 | 11.5 | 46.5 | 1.3 | 4.2 | 11.2 | 1.3 |
| Classroom Day3 Before | 27.2 | 6.0 | 42.8 | 1.5 | 3.9 | 17.5 | 1.1 |
| Classroom Day3 After | 31.0 | 4.6 | 37.7 | 1.7 | 5.4 | 18.4 | 1.2 |
| Sports field Day1 Before | 30.1 | 6.0 | 33.6 | 1.0 | 8.5 | 19.7 | 1.1 |
| Sports field Day1 After | 32.9 | 5.4 | 26.5 | 1.2 | 11.2 | 21.2 | 1.7 |
| Sports field Day2 Before | 28.8 | 8.5 | 30.6 | 1.2 | 11.3 | 18.5 | 1.0 |
| Sports field Day2 After | 38.7 | 5.9 | 23.0 | 1.1 | 10.6 | 18.9 | 1.8 |
| Sports field Day3 Before | 35.1 | 4.7 | 31.6 | 1.4 | 6.4 | 19.9 | 1.0 |
| Sports field Day3 After | 38.7 | 5.5 | 25.8 | 0.9 | 8.8 | 19.1 | 1.2 |
| Forest Day1 Before | 26.6 | 7.4 | 34.8 | 0.8 | 6.4 | 22.5 | 1.5 |
| Forest Day1 After | 32.4 | 6.4 | 29.7 | 1.0 | 9.7 | 18.4 | 2.3 |
| Forest Day2 Before | 32.0 | 7.1 | 32.1 | 1.4 | 7.4 | 18.5 | 1.5 |
| Forest Day2 After | 35.3 | 6.2 | 25.0 | 0.9 | 13.3 | 15.8 | 3.5 |
| Forest Day3 Before | 37.3 | 5.5 | 29.3 | 0.8 | 6.0 | 19.1 | 2.0 |
| Forest Day3 After | 36.6 | 6.5 | 21.0 | 0.7 | 17.3 | 14.6 | 3.3 |

**Figure S1** Ranked R^2^ values showing strength of difference between pairs of environmental exposure groups (i.e., classroom group ‘after’ exposure vs. classroom group ‘before’ exposure = CA – CB) from the (a.) weighted-UniFrac and (b.) unweighted-UniFrac PCoAs, respectively. C, ‘classroom’; F, ‘forest’; S, ‘sports field’; B, ‘before’ exposure; A, ‘after’ exposure. Grey bars represent no significant difference between pairs with α = 0.05, while black bars represent significant difference.


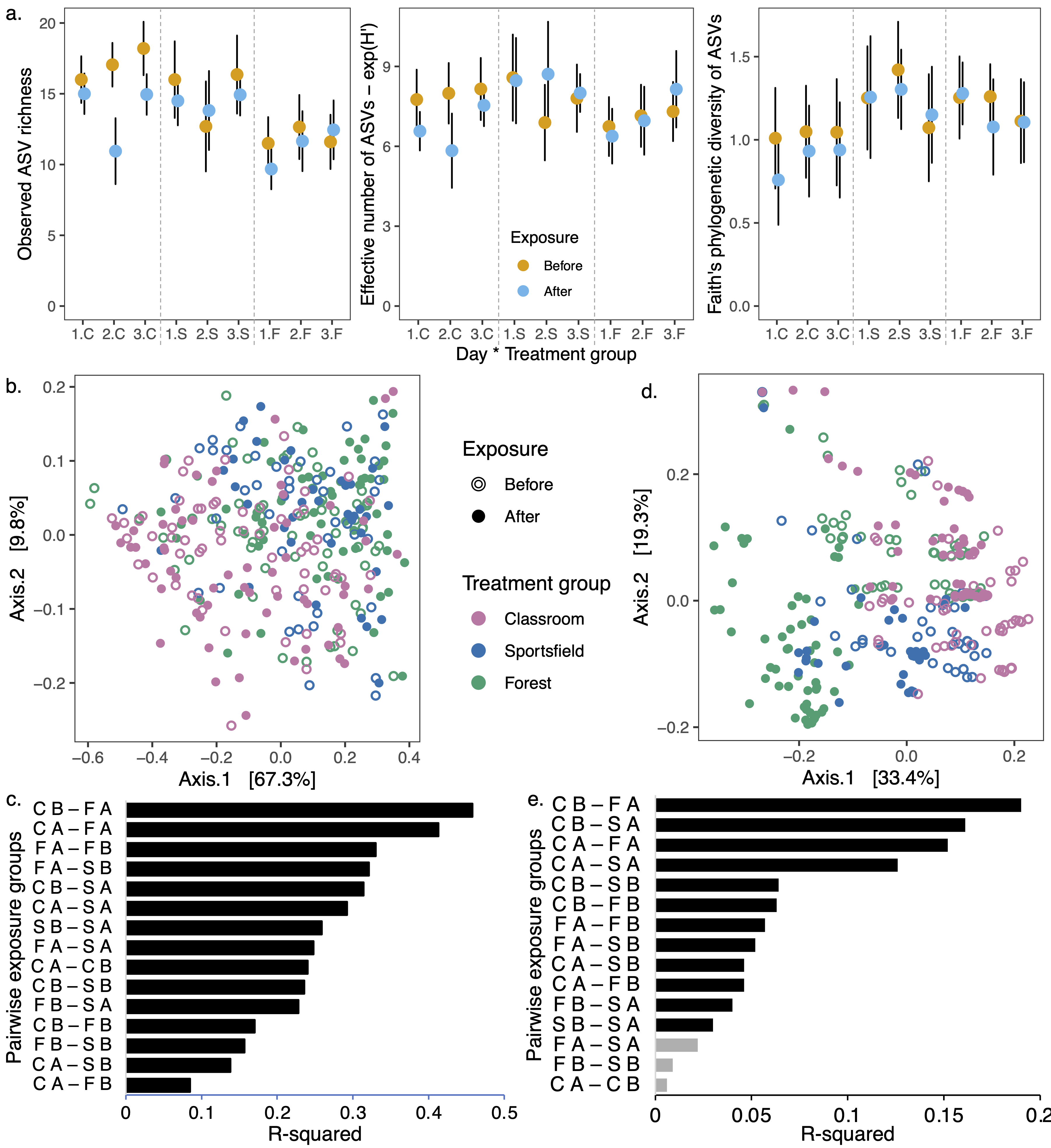


**Figure S2. Core bacterial ASV communities of children’s wrists before and after exposure to school yard environments. a.** Observed richness, effective number (exponent of Shannon’s diversity), and Faith’s phylogenetic diversity of ASVs are shown from the wrists of children exposed to three different school yard environments over three consecutive days. Points are means ± 95 % confidence interval. Significantly different pairs are listed in Table S1. 1, day 1; 2, day 2; 3, day 3; C, ‘classroom’; S, ‘sports field’; F, ‘forest’. **b. & d.** PCoA analyses of weighted-UniFrac and unweighted-UniFrac values, respectively, from all skin samples taken ‘before’ and ‘after’ outdoor exposure. **c. & e.** Bar plots ranking R^2^ values to show strength of difference between pairs of environmental exposure groups (i.e., ‘classroom’ group ‘after’ exposure vs. ‘classroom’ group ‘before’ exposure, CA – CB) from the weighted-UniFrac and unweighted-UniFrac PCoAs, respectively. C, ‘classroom’; S, ‘sports field’; F, ‘forest’; B, ‘before’ exposure; A, ‘after’ exposure. Grey bars represent no significant difference between pairs, black bars represent significant difference where *P* = 0.05.

**Figure S3. Alpha diversity of environmental samples from the exposure environments.** Shared letters indicate no significant difference between pairs from generalised linear models where *P* > 0.05.

**
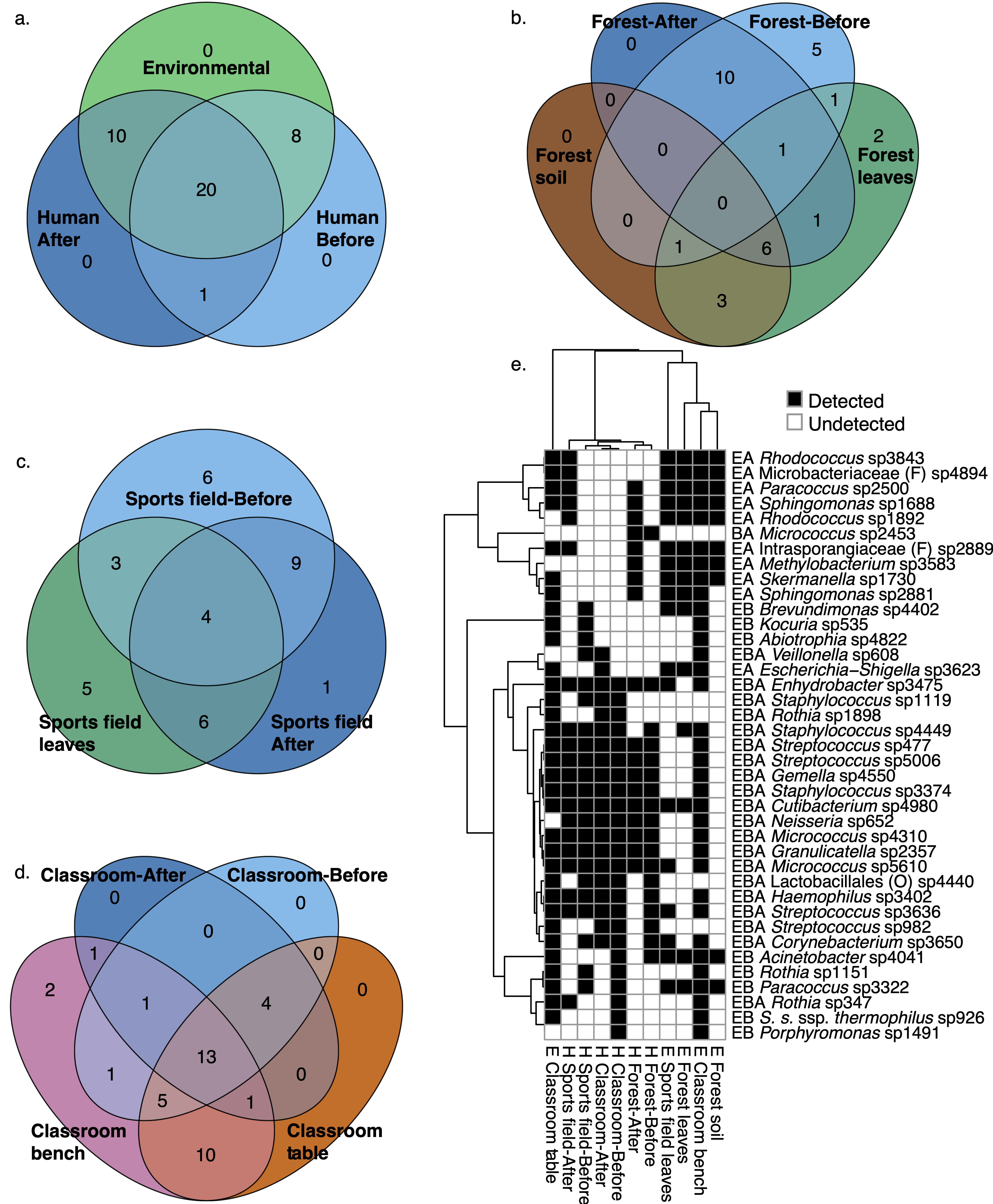
**

**Figure S4. Shared and unshared core bacterial community ASVs between human samples and environmental samples. a.** Total shared and unshared bacterial ASVs between environmental samples and human samples collected ‘before’ and ‘after’ exposure. **b.** Total shared and unshared bacterial ASVs between the forest environmental samples (soil and leaf surfaces) and human samples from the ‘forest’ treatment group collected ‘before’ and ‘after’ exposure. **c.** Total shared and unshared bacterial ASVs between the ‘sports field’ environmental samples (leaf surfaces) and human samples from the ‘sports field’ treatment group collected ‘before’ and ‘after’ exposure. **d.** Total shared and unshared bacterial ASVs between the ‘classroom’ environmental samples (bench tops and work tables) and human samples from the ‘classroom’ treatment group collected ‘before’ and ‘after’ exposure. **e.** A heatmap of detected community bacterial ASVs by sample type with clustering representing Pearson correlation between columns (samples) and between rows (ASVs). H and E on the x-axis represent human and environmental sample types, respectively. Y-axis lettering represents detection in environmental (E), human-before exposure (B), and/or human-after exposure (A) samples. All ASVs are named by their lowest identified taxonomic rank, and *S. s.* ssp. *thermophilus* is *Streptococcus salivarius* ssp. *thermophilus.*
